# The spectral transmission of ocular media suggests ultraviolet sensitivity is widespread among mammals

**DOI:** 10.1098/rspb.2013.2995

**Published:** 2014-04-07

**Authors:** R. H. Douglas, G. Jeffery

**Affiliations:** 1 Department of Optometry and Visual Science, City University London, Northampton Square, London EC1V 0HB, UK; 2 Institute of Ophthalmology, University College London, 11-43 Bath Street, London EC1V 9EL, UK

**Keywords:** vision, lens, transmission, mammal, ultraviolet sensitivity, retina

## Abstract

Although ultraviolet (UV) sensitivity is widespread among animals it is considered rare in mammals, being restricted to the few species that have a visual pigment maximally sensitive (*λ*_max_) below 400 nm. However, even animals without such a pigment will be UV-sensitive if they have ocular media that transmit these wavelengths, as all visual pigments absorb significant amounts of UV if the energy level is sufficient. Although it is known that lenses of diurnal sciurid rodents, tree shrews and primates prevent UV from reaching the retina, the degree of UV transmission by ocular media of most other mammals without a visual pigment with *λ*_max_ in the UV is unknown. We examined lenses of 38 mammalian species from 25 families in nine orders and observed large diversity in the degree of short-wavelength transmission. All species whose lenses removed short wavelengths had retinae specialized for high spatial resolution and relatively high cone numbers, suggesting that UV removal is primarily linked to increased acuity. Other mammals, however, such as hedgehogs, dogs, cats, ferrets and okapis had lenses transmitting significant amounts of UVA (315–400 nm), suggesting that they will be UV-sensitive even without a specific UV visual pigment.

## 1. Introduction

The range of wavelengths an animal perceives depends on the spectrum available in the environment, the degree to which this is transmitted though the ocular media and the visual pigments within the retina. The spectrum that humans see during the day, using three cone visual pigments absorbing maximally (*λ*_max_) at 420, 534 and 563 nm [1], spans approximately 400–700 nm. Adult humans are insensitive to shorter, ultraviolet (UV) wavelengths as these are absorbed by the lens [2–5] and hence never reach the retina.

The range of wavelengths visible to other animals is often very different from that of man due largely to their possession of visual pigments absorbing elsewhere in the spectrum. Many species, for example, possess visual pigments with *λ*_max_ below 400 nm, and the resultant UV-sensitivity is relatively widespread among invertebrates [6–8], birds, fish, reptiles and amphibians [9,10]. Among mammals, such UV-sensitive visual pigments are relatively rare and have only been described in some rodents [11–18], a mole [19], several marsupials [20–23] and some bats [24–27]. Such animals have lenses that, unlike those of humans, transmit short wavelengths well. UV sensitivity in mammals, in comparison to other animals, is thus thought to be the exception.

**Figure 1.**
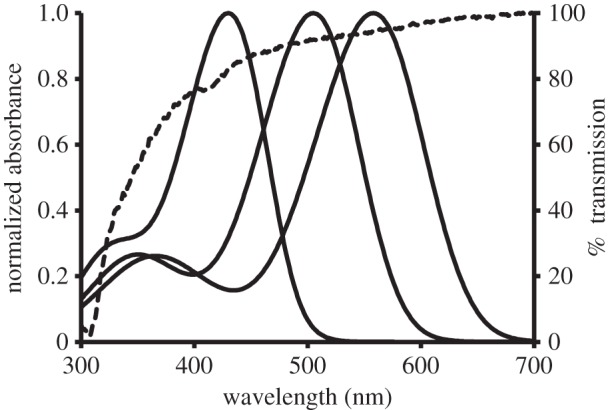
The absorption spectra of the visual pigments of the ferret and the spectral transmission of its lens. The absorption maxima of the visual pigments (rods—505 nm; cones—430 and 558 nm) are taken from Calderone & Jacobs [28] and the visual pigments templates of Govardovskii *et al.* [29] (solid lines) have been fitted to them using the methods described in Hart *et al*. [30]. The lens transmission (dotted line) is taken from this study. As all the visual pigments absorb significant amounts of UV radiation and the lens transmits in this part of the spectrum, the ferret is likely to perceive such short wavelengths.

Although visual pigments are usually characterized by their *λ*_max_, the wavelength range absorbed by them is in fact broad and displays a secondary absorption maximum in the UV (the *cis*-peak or β-band). Thus, all photoreceptors can potentially absorb significant amounts of UV and any animal with ocular media that are transparent to UV light will inevitably be sensitive to these wavelengths even if they do not possess a visual pigment with *λ*_max_ in this part of the spectrum [9] (figure 1).

Therefore, if the UV-absorbing lens of humans is removed following cataract surgery or traumatic injury, and not replaced by a UV-absorbing prosthetic, they report vivid and detailed vision in the UV [6,31–33]. Similarly, despite the absence of a visual pigment with *λ*_max_ in the UV, the reindeer retina responds electrophysiologically below 400 nm [34] and circadian rhythms in the Syrian hamster can be entrained by wavelengths below 400 nm [35–40], simply because they both have lenses that transmit significant amount of light below 400 nm rather than a visual pigment with *λ*_max_ in the UV. Likewise, a phyllostomid flower bat with a UV-transparent lens is able to respond behaviourally to UV even in scotopic conditions using the β-band of its rod pigment [41]. A degree of UV sensitivity in the absence of a photoreceptor absorbing maximally in this part of the spectrum is therefore not infrequent.

The spectral transmission of the ocular media (cornea, lens, aqueous and vitreous humour) at short wavelengths is determined by their structural components, thickness and any specific short-wave absorbing pigments they contain [42]. No structure will transmit significant amounts of light below about 300 nm owing to absorption by its nucleic acids and structural protein components, for example aromatic amino acids. With the exception of some fish corneas that absorb blue/green light [43–45], most corneas are thin and unpigmented, transmitting UV radiation down to around 300 nm. Aqueous and vitreous humours are similarly transparent. Lens transmission, however, is very variable. In some species, the lens lets through almost as much UV as the cornea, while in others it can remove all UV and some of the blue, appearing visibly yellow. Thus, although oil droplets in birds, for example, prevent short wavelengths from reaching the outer segments of some cones, and pigments such as the human macular pigment absorb blue light, the lens is the filter that determines the cut-off in the UV in almost all species (see the electronic supplementary material, S1).

Unfortunately, detailed information about the spectral transmission of most mammalian lenses is lacking. The only ones that have been examined are those that seem obviously interesting. At one extreme, the lenses of diurnal primates [5,46–49], tree shrews [50,51] and sciurid rodents [12,39,52–58] are various shades of yellow, removing all radiation below 420–470 nm. At the other extreme, species with visual pigments with *λ*_max_ in the UV have lenses maximally transparent to UV radiation, transmitting most light down to 320–340 nm [3,4,12,16,18,19,23,25,36,37,59–64]. However, little is known about the wavelengths transmitted by the lenses of mammals between these two extremes. Although the lenses of some have been reported as containing no short-wavelength-absorbing pigment [54], reliable quantitative transmission data from intact lenses are only available only for the Syrian hamster (*Mesocricetus auratus*) [35–39], pig (*Sus scrofa*) [4], rabbit (*Oryctolagus cuniculus*) [3,65–68] and reindeer (*Rangifer tarandus*) [34].

Here, we examine the spectral transmission of the lenses of 38 mammalian species belonging to 25 families in nine orders, most never examined before, and show a variety of degrees of shortwave transmission. Perhaps surprisingly, many let through significant amounts of shortwave radiation, suggesting that a degree of UV sensitivity is widespread among mammals.

## 2. Material and methods

Animals were obtained from various sources such as abattoirs, zoos, veterinary practices and scientific establishments (see Acknowledgements). They had either been used for other scientific procedures, sacrificed for food production, died naturally or were put down owing to injury or illness. No animals were killed specifically for this project. Eyes were obtained either immediately following death, or soon thereafter, and were either used immediately or frozen dry for several days before thawing. Variable numbers of lenses were available for each species and in four species a range of lens sizes/ages were examined (see table 1 for details).

**Table 1.** Summary of mammalian lenses examined ranked by the amount of UVA they transmit. ‘50%T’ is the wavelength at which the lens transmits 50% of the incident illumination. ‘%UVA transmitted’ is a measure of the proportion of light between 315 and 400 nm that is transmitted by the lens (see the electronic supplementary material, S2). For most species, lens transmission and axial diameter (pathlength) varied little between individuals and averages are shown. Where there were significant differences between individuals, ranges are given. Where the transmission of the lens varied with lens size/age, the % UVA on the retina was calculated using specific ages/lens sizes as described in footnotes.

order	family	species	number of lenses	pathlength (mm)	50%T (nm)	%UVA transmitted
Rodentia	Muridae	mouse (*Mus musculus*)	29	1.9–2.8	313–337	81.4^a^
Rodentia	Muridae	black rat (*Rattus rattus*)	11	3.7–5.2	317–372	80.5^b^
Erinaceomorpha	Erinaceidae	hedgehog (*Erinaceus europaeus*)	4	3.0	326	65.5
Carnivora	Canidae	dog (*Canis lupus familiaris*) (labrador)	2	5.0	335	61.3
Chiroptera	Pteropodidae	Livingstone's fruit bat (*Pteropus livingstonii*)	4	5.0–6.0	332–422	60.8^c^
Carnivora	Felidae	cat (*Felis catus)*	6	7.0	345	58.9
Carnivora	Mustelidae	ferret (*Mustela putorius furo*)	4	3.9	344	56.1
Rodentia	Muridae	brown rat (*Rattus norvegicus*)	2	4.2	339	55.8
Artiodactyla	Giraffidae	okapi (*Okapia johnstoni*)	2	7.0	355	53.4
Artiodactyla	Suidae	pig (*Sus scrofa*)	5	5.5	375	43.6
Rodentia	Caviidae	guinea pig (*Cavia porcellus*)	11	3.7	377	34.6
Carnivora	Ailuridae	red panda (*Ailurus fulgens*)	1	5.8	386	30.2
Rodentia	Sciuridae	flying squirrel (*Glaucomys volans*)	2	4.9	423	29.3
Chiroptera	Pteropodidae	Rodrigues flying fox (*Pteropus rodricensis*)	1	4.8	388	28.1
Artiodactyla	Cervidae	reindeer (*Rangifer tarandus*)	5	10.1	384	26.5
Artiodactyla	Cervidae	pudú (*Pudu puda*)	2	7.0	386	25.0
Artiodactyla	Bovidae	cattle (*Bos primigenius*)	8	11.1	384	22.1
Artiodactyla	Bovidae	sheep (*Ovis aries*)	4	7.7	393	15.2
Rodentia	Dasyproctidae	agouti (*Dasyprocta punctata*)	1	6.1	406	15.0
Lagomorpha	Leporidae	rabbit (*Oryctolagus cuniculus*)	2	6.7	392	12.7
Artiodactyla	Tragulidae	java mouse deer (*Tragulus javanicus*)	2	9.0	403	12.4
Artiodactyla	Bovidae	Arabian oryx (*Oryx leucoryx*)	1	10.3	400	8.5
Artiodactyla	Camelidae	alpaca (*Vicugna pacos*)	5	10.2	405	6.0
Perissodactyla	Equidae	horse (*Equus ferus caballus*)	1	12.0	416	4.6
Primates	Cebidae	squirrel monkey (*Saimiri sciureus sciureus)*	2	4.6	420	2.8
Primates	Lemuridae	ring-tailed lémur (*Lemur catta*)	1	6.5	425	2.0
Carnivora	Herpestidae	meerkat (*Suricata suricatta*)	3	2.4–3.4	420–436	1.7^d^
Primates	Callitrichidae	marmoset (*Callithrix jacchus*)	1	3.0	427	0.9
Artiodactyla	Bovidae	lowland anoa (*Bubalus depressicornis*)	1	8.0	478	0.6
Rodentia	Sciuridae	ground squirrel (*Urocitellus richardsonii*)	2	3.1	462	0.6
Primates	Cercopithecidae	macaque (*Macaca fascicularis*)	5	3.3	424	0.5
Primates	Atelidae	red-faced spider monkey (*Ateles paniscus*)	1	3.8	438	0.4
Primates	Callitrichidae	golden lion tamarin (*Leontopithecus rosalia*)	1	3.0	441	0.4
Scandentia	Tupaiidae	Tree shrew (*Tupaia glis*)	1	3.2	435	0.3
Primates	Lemuridae	Alaotran gentle lemur (*Hapalemur alaotrensis*)	1	5.9	425	0.3
Rodentia	Sciuridae	grey squirrel (*Sciurus carolinensis*)	2	3.6	441	0
Rodentia	Sciuridae	prairie dog (*Cynomys ludovicianus*)	7	3.6	463	0
Primates	Cebidae	capuchin (*Cebus apella*)	1	3.9	426	0

^a^Aged 69–72 days with lens pathlength 2.2 mm.

^b^Pathlength 3.8 mm.

^c^Pathlength 5.0 mm.

^d^Pathlength 3.4 mm.

Lenses, and usually corneas, were removed from the eye, briefly rinsed in phosphate-buffered saline (PBS) and mounted in purpose-built holders in air in front of an integrating sphere within a Shimadzu 2101 UVPC spectrophotometer. Vitreous humour was also removed from the eyes of some animals with a syringe and placed in a standard quartz cuvette within the same apparatus. Transmission at 700 nm was set to 100% and ocular media scanned at 1 nm intervals from 300 to 700 nm.

To determine the effect of freezing on lens transmission, three fresh bovine lenses were scanned soon after death, frozen in air at −25°C for 4 days, thawed and rescanned.

The pigments responsible for lens pigmentation were also extracted and spectrally characterized for six species (see the electronic supplementary material, S4).

## 3. Results

Although the cornea and vitreous humour were not examined in all species, when they were, in line with previous observations [42], the lens always removed more short-wavelength radiation than either the cornea or the vitreous (see the electronic supplementary material, S1).

**Figure 2.**
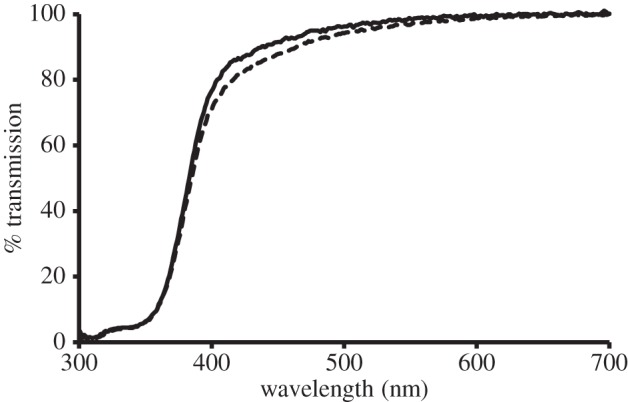
Average spectral transmission of three bovine lenses before (solid line) and after (dashed line) four days of freezing.

Freezing had no significant effect on lens transmission, allowing data from both fresh and previously frozen lenses to be compared (figure 2).

The spectral transmission of the lenses of some of the species studied here had been examined previously; pig [4], tree shrew (*Tupaia glis*) [50], rabbit [3,65–68], mouse (*Mus musculus*) [3,4,63,64], brown rat (*Rattus norvegicus*) [3,4,12,36,37,60], grey squirrel (*Sciurus carolinensis*) [54,58], prairie dog (*Cynomys ludovicianus*) [55–56], flying squirrel (*Glaucomys volans*) [56], marmoset (*Callithrix jacchus*) [49], squirrel monkey (*Saimiri sciureus sciureus*) [46] and macaque (*Macaca fascicularis*) [48]. Our data for these species agreed with the previously published spectra. They are presented here to validate our method, facilitate direct comparison with novel data and allow further analysis.

**Figure 3.**
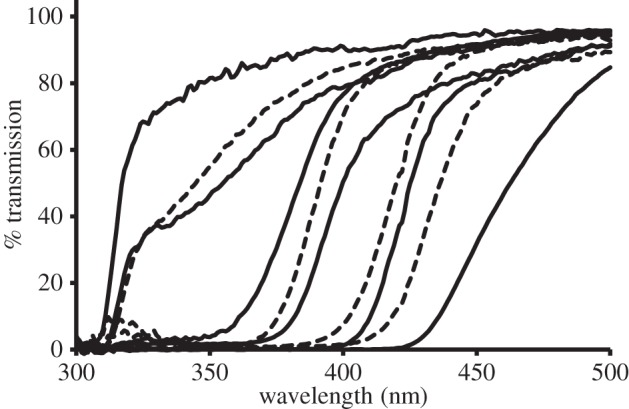
Representative average spectral transmission curves at short wavelengths of the lenses from 10 mammalian species. Most curves are the averages of all available lenses. However, for the black rat and meerkat individuals of a variety of lens sizes were scanned; the data shown for the two species are for young and old animals, respectively. From left to right at 50% transmission they are (*n*, lens axial diameter in millimetres); young black rats (2, 3.8), cat (6, 7.0), okapi (2, 7.0), cattle (8, 11.1), rabbit (2, 6.7), Arabian oryx (1, 10.3), squirrel monkey (2, 4.6), Alaotran gentle lemur (1, 5.9), adult meerkat (1, 3.4) and prairie dog (7, 3.6). All scans were zeroed at 700 nm.

The spectral transmission of representative lenses is shown in figure 3 and equivalent scans for all species are shown in the electronic supplementary material, S3. The spectral properties of the mammalian lenses examined ranged from those in young murid rodents as well as juvenile hedgehogs, which transmitted large amounts of UV radiation (50% transmission 310–320 nm), to those of primates, sciurid rodents, meerkats and tree shrews that were visibly yellow and prevented UV radiation from reaching the retina (50% transmission 424–465 nm). All other mammals had lenses whose spectral transmission lay between these two extremes (table 1 and figure 3; also see the electronic supplementary material, figure S3a–d).

The degree of UV radiation transmitted by the lens is traditionally expressed as the wavelength of 50% transmission. However, this measure can be misleading as the short-wavelength cut-off is sometimes steep, but at other times gentle. Thus, although the flying squirrel and the macaque both have a similar wavelength of 50% transmission (423–424 nm), their spectral characteristics at short wavelengths are in fact quite different (see the electronic supplementary material, figure 3*c*). A better indication of the potential for UV vision is given by the proportion of UVA (315–400 nm) that is transmitted by the lens (table 1; see the electronic supplementary material, S2).

**Figure 4.**
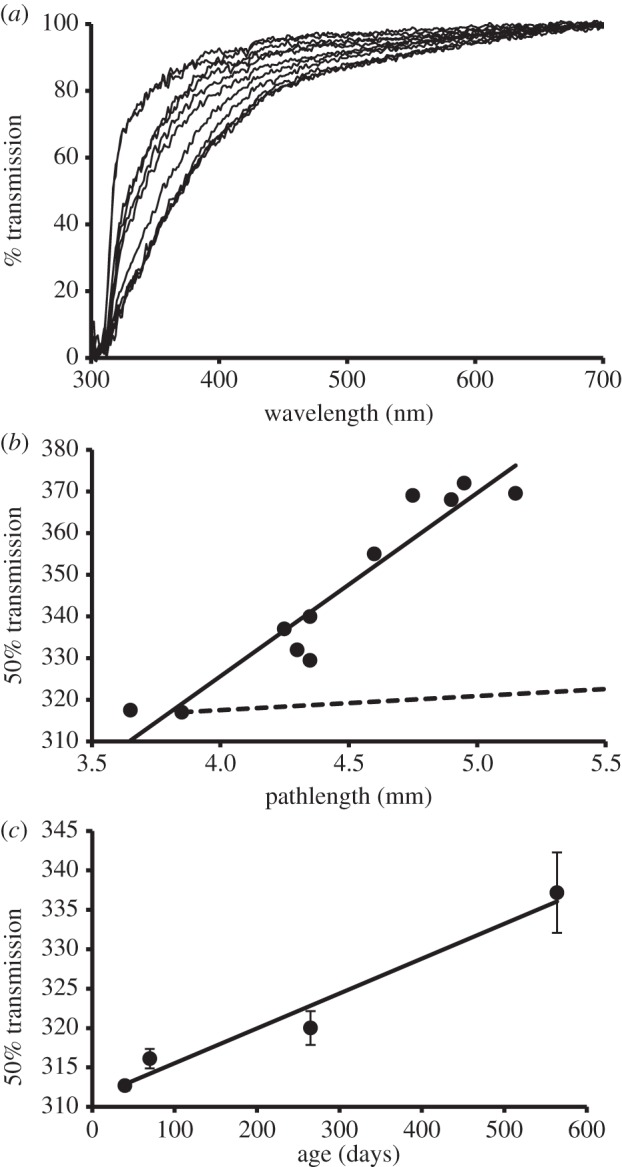
Lens transmission as a function of lens size/age in rodents. (*a*) Spectral transmission of 11 black rat (*R. rattus*) lenses ranging in axial length between 3.7 and 5.2 mm. (*b*) Wavelength of 50% transmission as a function of lens size for all the lenses shown in (*a*). The data are fit by *y* = 43.992*x* + 149.68 (*R*² = 0.9062). The dashed line is an approximation of the relationship expected if pathlength were the only factor affecting transmission. (*c*) Average wavelength of 50% lens transmission (±1 s.d.) of mice (*M. musculus*) of known age; 40 (*n* = 3), 70 (*n* = 8), 265 (*n* = 4) and 564 (*n* = 6) days. The data are fit by *y* = 0.0443*x* + 311.1 (*R*² = 0.9634).

For four species (*Pteropus livingstonii*, *Rattus rattus*, *Mus musculus* and *Suricata suricatta*), lenses from a range of ages/sizes were available and exhibited decreased short-wavelength transmission in older/larger lenses. Data are shown only for the rat and mouse (figure 4) as the largest number of differently sized lenses were available for them. Similar trends were shown by lesser numbers of Livingston's bats (*n* = 4) and meerkats (*n* = 3).

The eyes of the Alaotran gentle lemur and the ring-tailed lemur, apart from containing a distinctly yellow coloured lens, on dissection revealed a bright yellow substance within the eye. Strepsirrhine primates are known to have yellow riboflavin-based tapeta [69], which is almost certainly the source of this pigmentation.

A pigment absorbing maximally at 357–369 nm was extracted from the lenses of six species with yellow lenses and identified as 3-hydroxykynurenine glucoside in two (see the electronic supplementary material, S4).

## 4. Discussion

### (a) Diversity of ultraviolet transmission by mammalian lenses

As expected, the transmission of short wavelengths by the mammalian lens varies considerably between species. At one extreme, as has previously been reported, species with visual pigments absorbing maximally in the UV–for example murid rodents–transmit up to 80% of UVA radiation (figure 3 and table 1; see also the electronic supplementary material, S3). The most UV-transparent lens observed outside of such animals belongs to the European Hedgehog (*Erinaceus europeus*; see the electronic supplementary material, figure S3b). Interestingly, preliminary evidence suggests that the closely related Southern white-breasted hedgehog (*Erinaceus concolor*) may in fact possess a visual pigment with *λ*_max_ below 400 nm (M. Glösmann 2013, personal communication). In stark contrast, the lenses of mature diurnal primates, sciurid rodents, tree shrews and meerkats contain a pigment absorbing maximally around 360–370 nm (see the electronic supplementary material, S4). Consequently, they absorb all UV radiation and a considerable amount of blue light, appearing visibly yellow (figure 3 and table 1; see also the electronic supplementary material, S3). Although it is well established that sciurid rodents and diurnal primates have yellow lenses, we have expanded the number of species within these orders known to have such lenses, and their presence in meerkats is a novel observation.

However, most mammals appear to have lenses between these two extremes that transmit variable amounts of shortwave radiation (figure 3 and table 1; see also the electronic supplementary material, S3). Relatively large lenses like those of the horse (*Equus ferus caballus*), alpaca, oryx, anoa (*Bubalus depressicornis*) and mouse deer remove the vast majority of the UV, although as they remove little visible radiation, they do not appear obviously yellow. On the other hand, animals such as the cat, dog, ferret and okapi have lenses that transmit only slightly less UVA than those of some murid rodents (figure 3 and table 1; see also the electronic supplementary material, S3).

As expected, species that are at least partially nocturnal generally have lenses transmitting UV, while those that are mainly diurnal prevent such wavelengths reaching the retina. For example, while diurnal sciurid rodents have yellow lenses that remove all UV, the nocturnal flying squirrel has a clear lens [56] that transmits significant amounts of UVA (table 1; see also the electronic supplementary material, figure S3c). However, such a demarcation is not absolute and some animals, like the okapi, can be exposed to relatively high amounts of daylight while having lenses that transmit relatively large amounts of UV.

### (b) Lens transmission indicates that a degree of ultraviolet sensitivity may be widespread among mammals

Only species with UV-transparent lenses and a visual pigment with *λ*_max_ below 400 nm are usually considered UV sensitive. However, as all visual pigments have a degree of photosensitivity at such short wavelengths, an animal with a lens transmitting UV radiation will inevitably be sensitive in this part of the spectrum, even in the absence of a specific UV-absorbing visual pigment.

Species are ranked according to the amount of UVA transmitted by the lens in table 1. A previous study has shown that the reindeer, whose lens transmits 26.5% of UVA and which does not have a visual pigment with *λ*_max_ below 400 nm, nevertheless responds electrophysiologically to 372 nm light [34]. It therefore seems likely that species with similar or more UV lens transmission, such as cattle, pig, ferret, dog, okapi and cat, for example, will also be sensitive at these short wavelengths (table 1).

The realization that many mammals have some UV sensitivity may be important for understanding aspects of their behaviour as they could be responding to visual signals undetectable to humans. It may also have implications for the lighting conditions of captive and domestic species. On the one hand, some UV may be required for normal behaviour, while on the other, excessive UV exposure might put species with UV-transparent ocular media at increased risk of retinal damage (see below).

### (c) The nature of ultraviolet sensitivity without a *λ*_max_ at short wavelengths

It might be argued that UV perception not mediated by a visual pigment with *λ*_max_ in this part of the spectrum is in some way not ‘real’ UV sensitivity. However, nobody questions a human's ability to see red light with a wavelength of 700 nm, despite the fact that our long-wavelength sensitive cone absorbs maximally at wavelengths more than 100 nm removed from this.

Although all visual pigments can absorb UV radiation, the way the signals generated by the photoreceptors to such wavelengths are processed is not known. Thus, animals without a visual pigment with *λ*_max_ in the UV will probably be unable to distinguish UV as a separate colour. Aphakic humans, for example, report UV as appearing like a desaturated (whitish) blue-violet [6].

The extent of UV sensitivity in animals with UV-transparent ocular media but without a specific UV visual pigment is also uncertain. However, it is probable that such animals will be less sensitive at these wavelengths than species that do have such a visual pigment, although the photopic sensitivity of aphakic humans [6] and reindeer [34] to UV light is surprisingly high. Such sensitivity will be influenced by several factors. For example, as the absorptance spectrum of a visual pigment is influenced by pigment density, the degree of UV sensitivity will depend in part on the length of a species’ outer segments and the presence of a tapetum (which would effectively double the pathlength of the outer segment). Furthermore, the nature of the interactions between the different photoreceptor types at short wavelengths and the effectiveness of short wavelengths at triggering the transduction cascade, neither of which are known, will also influence the degree of shortwave sensitivity.

### (d) Function of ultraviolet sensitivity in mammals

It is tempting to seek a specific function for UV sensitivity, although similar questions are rarely asked about other parts of the spectrum. The functions proposed include; mate choice, ‘secret’ intraspecific communication, navigation, prey detection and foraging. However, UV light is little different from other parts of the spectrum and its perception need have no specific function beyond simply extending the spectral range of the animal and improving its sensitivity. Indeed, although UV has a role to play in both foraging and mate choice in birds, longer wavelengths have been shown to be more important [70,71]. Although in some instances, UV may have a specific function, such as increasing the visibility of the white fur of predatory polar bears within a snowy landscape for reindeer [34] or enhancing the visibility of urine trails for rodents [15,16], UV is normally just a part of a wider spectrum of wavelengths all of which are important for an animal's behaviour.

Perhaps, the reason why there is a tendency to attribute some special importance to UV sensitivity is simply that humans are not able to see it [72].

### (e) What is the function of preventing ultraviolet radiation from reaching the retina?

Shortwave-sensitive visual pigments come in two forms: violet-sensitive or UV-sensitive (UVS). Molecular evidence suggests the UVS visual pigments are the ancestral form [73]. Logically therefore, UV-transmitting lenses are also ancestral and animals must have been subjected to selective pressure to lose both UVS visual pigments and UV lens transmission. Therefore, rather than seeking a specific function for UV vision in mammals, it might be more pertinent to ask, what is the function of animals having lenses that prevent short wavelengths reaching the retina? Blocking UV could be either protective or an aid to spatial resolution [42]. These different functions are by no means mutually exclusive and both would explain the presence of UV-absorbing lenses in mainly diurnal animals.

Removing short wavelengths, especially in long-lived diurnal species, could protect the retina as the degree of retinal light damage is considerably increased at shorter wavelengths [74]. There is some experimental evidence for such a function. For example, when the UV-absorbing lenses of grey squirrels were removed, the retinae of these eyes suffered more retinal damage than intact companion eyes [75]. It has therefore been suggested that the reason nocturnal rodents, for example, can have UV-sensitive visual pigments (and a UV-transparent lens) is that they are relatively short-lived and habitually exposed to low light levels. However, the lens cannot have a protective role in all species. The reindeer, for example, lives in an extremely UV-rich environment and can reach ages of up to 20 years, yet it seems to suffer no ill effects from allowing UV to reach the retina [34]. Similarly, some UV-sensitive parrots can live to be over 50 years old with no apparent damage [76]. Either species such as reindeer and parrots have mechanisms to prevent the harmful effects of UV, or some species are particularly sensitive to its deleterious consequences.

Short-wave absorbing filters will also increase image quality as both the degree of Rayleigh scatter and chromatic aberration are increased in this part of the spectrum [52], although such a function is difficult to prove experimentally. Interestingly, species such as diurnal primates and sciurid rodents, whose lenses do remove short wavelengths, either have a large proportion of cones (more than 20%) within the retina and/or areas of very high cone density (more than 100 000 cones mm^−2^; see the electronic supplementary material, table S5), which is consistent with a function of such filters being to increase image quality. Interestingly, the same argument has very recently been suggested to account for the UV-absorbing ocular media of diurnal raptors, which have extremely high visual acuity to facilitate the capture of moving prey on the wing [77].

For species active that are at night, on the other hand, the primary visual requirement is high absolute sensitivity rather than spatial acuity, which will be facilitated by a UV-transparent lens. Such animals generally have a lower proportion of cones in their retina and no areas of increased cone density, but often have areas of increased rod density consistent with maximizing absolute sensitivity (see the electronic supplementary material, table S5).

### (f) Size(age)-related changes in lens transmission

It is not possible to characterize the spectral transmission of a species’ lens by a single curve, as it will inevitably change as a function of lens size. In some species, such as man, the lens grows throughout life [78], and its size can be used to age the animal [79]. In other species, lens growth levels off in older animals [80]. Generally, as shown by the four species in this study for whom a range of lens sizes were available (*P. livingstonii*, *R. rattus*, *M. musculus* and *S. suricatta*), the relative transmission of short wavelengths decreases with increased lens size and age (figure 4).

Some age-related change in lens transmission is an inevitable consequence of increased pathlength in older animals. An approximate indication of the effect of lens size on spectral transmission can be obtained by squaring the transmission spectrum of a small lens to give a theoretical curve for a lens twice the diameter [81,82]. For both the rat (figure 4*b*) and mouse (data not shown), increased size is insufficient to account for the decreased transmission observed. The causes for the frequently described age-related yellowing of the lens of primates [5,83] are complex [78] but are in part the result of the attachment of the major tryptophan-derived, short-wave absorbing lens pigment (see the electronic supplementary material, S4) to lens proteins [47,84]. The proximate causes of the decreased shortwave transmission in other species, for example those described here, that cannot be the result of a simple increase in pathlength, are unclear.

It seems likely that such age-related changes in lens transmission are the inevitable result of both increased lens size and light exposure. Nonetheless, they might protect the retina of older animals from the harmful UV radiation and, for example, slow the rate of photoreceptor loss.

## Supplementary Material

Supplementary Material
