# Supplementary material for: The spectral transmission of ocular media suggests ultraviolet sensitivity is widespread among mammals
Source: Proc Biol Sci. 2014 Apr 7;281(1780):20132995. doi: 10.1098/rspb.2013.2995 (PMC4027392; doi:10.1098/rspb.2013.2995)
Supplement: Supplementary Material [file rspb20132995supp1.docx]

**Supplementary Material for:**

**The spectral transmission of ocular media suggests ultraviolet sensitivity is widespread among mammals**

R.H. Douglas^1,*^ & G. Jeffery^2^

^1^Department of Optometry & Visual Science, City University London, Northampton Sq, London EC1V 0HB, UK

^2^Institute of Ophthalmology, University College London, 11-43 Bath Street, London EC1V 9EL, UK

**Contents**

**S1** Comparison of lens transmission to that of the aqueous humour and cornea **page 2**

**S2** Calculation of the proportion of UVA transmitted by the lens **page 3**

**S3** Spectral transmission of lens from all species examined **pages 4-6**

**S4** Extraction and identification of lens pigments **pages 7-8**

**S5** Correlation between lens transmission and retinal morphology **pages 9-10**

**S6** Additional references **pages 11-13**

**S1. Comparison of lens transmission to that of the aqueous humour and cornea.**

Although lenses were examined in all 38 species, due usually to the small size of the eye, corneas were only scanned from 30 and the vitreous in only 2. In all animals tested the lens always removed more short wavelength radiation than either the cornea or the vitreous.

**Supplementary figure 1 Spectral transmission of bovine ocular media**. The small irregularities in the transmission of the cornea and vitreous around 400nm are caused by contamination with blood.

**S2. Calculation of the proportion of UVA transmitted by the lens**

**Supplementary figure S2 The proportion of UVA transmitted by the lenses of four species of mammal.** This represents the area under the curve for each species within the rectangle shown expressed as a ercentage of the area of the entire rectangle. Equivalent values for all species are shown in table 1.

**S3. Spectral transmission of lenses from all species examined**

**Supplementary figures 3a-d Spectral transmission of all mammalian lenses used in ths study**.

In most cases average scans of all available lenses are shown. However, in 4 species lenses of a variety of sizes were scanned whose spectral transmission differed (black rat, mouse, Livingstone’s bat and meerkat). For these species the transmission of a specific lens size is shown. The legends indicate the number of lenses scanned that make up each curve and the average pathlength of the lens. The data for 5 lenses (shown in figs S3b&c) came from a previous unpublished study in which lens transmission was zeroed at 500nm, while for all other lenses transmission at 700nm was set at zero. Zeroing at 500nm will steepen the curve somewhat and shift the wavelength of 50% transmission slightly towards shorter wavelengths.

**S4. Extraction and identification of lens pigments**

The lenses of grey squirrels and various primates (human, baboon, rhesus monkey, squirrel monkey & bush baby) contain pigments with absorption maxima around 360-370nm [1,2].

The nature of the pigment responsible for the visibly yellow colouration of lenses in six of the species studied here was investigated by homogenising lenses in distilled water and centrifuging (4ºC, 100,000g) the homogenate for 10-30 minutes and scanning the supernatant . In some species the supernatant was further purified by ultrafiltration under nitrogen using Amicon YM2 membranes with pore size 100da fitted to a 50ml Amicon cell, before scanning.

We extracted pigments with absorption spectra similar to those described by Cooper & Robson [1,2] in the lenses of four more primates (macaque, marmoset, capuchin, ringtailed lemur), prairie dogs and meerkats (Fig S4).

**Supplementary figure 4 Normalised absorbance spectra of aqueous extracts of the yellow lenses from 6 species of mammals**

The pigments of primate and grey squirrel lenses have been identified as kynurenine-related tryptophan derivatives [3-11]. Similar pigments have been characterised in ground squirrels [12] and even some fish [13,14].

To confirm this identification in the macaque and marmoset their lyophilised lens pigment extracts were prepared for isocratic reverse-phase high performance liquid chromatography (HPLC) by redissolving ca. 5mg in 1ml 20% methanol and filtering through a C-18 Cep Pak cartridge. A Whatman 10 ODS-3 semi-preparative column (250x9.4mm internal diameter) protected by a Bondapak C-18 pre-column was fitted to the system. A mobile phase of 20% methanol and 0.1% acetic acid (filtered and degassed prior to use) was used as detailed by Dunlap et al. [15], with a flow rate of 2ml/min. Filtrate absorbance was monitored on a Knauer variable wavelength detector at 340nm and recorded on a Shimadzu C-R6A Chromatopac recorder. Up to 0.5ml of sample was injected into the system and any peaks were collected, pooled and lyophilised. Dried purified samples were redissolved in 1ml 20% methanol and scanned on a Shimadzu UV-250 spectrophotometer. Pigments were identified by injecting an equal volume of a known standard with the sample and monitoring any increase in absorbance at the retention time of the standard.

The lens pigments of both the marmoset and macaque co-chromatographed

with 3-hydroxykynurenine glucoside, the major pigment in human lenses, suggesting that perhaps all mammals with yellow lenses contain 3-hydroxykynurenine or a modified derivative of this molecule.

**S5 Correlation between lens transmission and retinal morphology**

| **Species** | **% UVA transmitted** | **% cones** | **Peak cone density (mm^2^)** | **Peak rod density (mm^2^)** |
| --- | --- | --- | --- | --- |
| Mouse (*Mus musculus*) | 81.4^a^ | 2.8^16^ | 15,170^16^ | 505,208^16^ |
| Black rat (*Rattus rattus*) | 80.5^b^ | 1.0-2.0^17^ | - | - |
| Hedgehog (*Erinaceus europaeus*) | 65.5 | 1.5-2.7^18^ | 6,500^18^ | 350,000^18^ |
| Dog (*Canis lupus familiaris*) | 61.3 | - | 23,080^19^ | 501,000^19^ |
| Livingstone’s fruit bat (*Pteropus livingstonii*) | 60.8^c^ | *0.4-0.6^20^* | *11,000^20^* | *800,000^20^* |
| Cat (*Felis catus)* | 58.9 | - | 26,500^21^ | 460,000^21^ |
| Ferret (*Mustela putorius furo*) | 56.1 | - | 26,183^22^ | 349,428^22^ |
| Brown rat (*Rattus norvegicus*) | 55.8 | 0.9^23^ | - | - |
| Okapi (*Okapia johnstoni*) | 53.4 | - | *-* | - |
| Pig (*Sus scrofa*) | 43.6 | 11.1^24^ | 39,000^24^ | 151,000^24^ |
| Guinea pig (*Cavia porcellus*) | 34.6 | 8.0-17^25^ | 30,000^25^ | 290,000^25^ |
| Red panda (*Ailurus fulgens*) | 30.2 | - | - | - |
| Flying squirrel (*Glaucomys volans*) | 29.3 | - | - | - |
| Rodrigues flying fox (*Pteropus rodricensis*) | 28.1 | *0.4-0.6^20^* | *11,000^20^* | *800,000^20^* |
| Reindeer (*Rangifer tarandus*) | 26.5 | - | *-* | - |
| Pudú (*Pudu puda.*) | 25.0 | - | 20,600^26^ | - |
| Cattle (*Bos premigenius*) | 22.1 | - | 21,000^27^ | 250,000^27^ |
| Sheep (*Ovis aries*) | 15.2 | 8.3^28^ | 28,000^28^ | 270,000^28^ |
| Agouti (*Dasyprocta punctata*) | 15.0 | *10-19^29^* | *14,000^29^* | *64,000^29^* |
| Rabbit (*Oryctolagus cuniculus*) | 12.7 | 3.5-4.5^30^ | 16,000^30^ | 308,000^30^ |
| Java mouse deer ( *Tragulus javanicus*) | 12.4 | - | 19,900^26^ | - |
| Arabian oryx (*Oryx leucoryx*) | 8.5 | - | 32,700^26^ | - |
| Alpaca (*Vicugna pacos*) | 6.0 | - | *-* | - |
| Horse (*Equus ferus caballus*) | 4.6 | <10^31^ | 20,000^32^ | 170,000^33^ |
| Squirrel monkey (*Saimiri sciureus sciureus*) | 2.8 | *9.3^34^* | *138,021^34^* | *37,939^34^* |
| Ring-tailed lémur (*Lemur catta*) | 2.0 | - | - | - |
| Meerkat (*Suricata suricatta*) | 1.7^d^ | *-* | - | - |
| Marmoset (*Callithrix jacchus*) | 0.9 | 24.3^34^ | 132,813^34^ | 79,102^34^ |
| Lowland anoa (*Bubalus depressicornis*) | 0.6 | - | - | - |
| Ground squirrel (*Urocitellus richardsonii*) | 0.6 | *85.5^35^* | *49,550^35^* | *13,000^35^* |
| Macaque (*Macaca fascicularis*) | 0.5 | 6.1^36^ | 210,000^36^ | *184,000^37^* |
| Spider monkey (*Ateles paniscus*) | 0.4 | - | - | - |
| Golden lion tamarin (*[Leontopithecus](http://en.wikipedia.org/wiki/Leontopithecus) rosalia*) | 0.4 | *-* | *-* | *-* |
| Alaotran gentle lemur (*Hapalemur alaotrensis*) | 0.3 | - | - | - |
| Treeshrew (*Tupaia glis*) | 0.3 | *94-96^38^* | *36,000^38^* | *3,500^38^* |
| Grey squirrel (*Sciurus carolinensis*) | 0.0 | 64.0^39^ | - | - |
| Prairie dog (*Cynomys ludovicianus*) | 0.0 | 89.3^39^ | - | - |
| Capuchin (*Cebus apella*) | 0.0 | 7.9^34^ | 164,062^34^ | 174,967^34^ |

**Supplementary table S5 Retinal parameters of species ranked in order of the amount of UVA that penetrates the lens.**

Where possible retinal data from the same species used in this study are shown. If these were unavailable data were taken from species within the same genus (these values are shown in italics). In several instances more than 1 author produced retinal data for the same species. In such cases the most recent or comprehensive was used. There were some differences between various studies on the same species but not significant enough to affect the overall conclusions. The shaded values indicate arbitrary thresholds of more than 20% cones or a peak density of over 100,000 cones/mm^2^, which are indicative of a retina specialised for high spatial acuity. Where the transmission of the lens varied with age, the % UVA on the retina was calculated using specific ages/lens sizes as follows; a - aged 69-72 days with lens pathlength 2.2mm, b – pathlength 3.8mm, c – pathlength 5.0mm, d – pathlength 3.4mm.

**S6 Additional references**

1. Cooper GF, Robson JG 1969a The yellow colour of the lens of the grey squirrel (*Sciurus carolinesis leucotis*). *J. Physiol. London* **203**, 403–410.

2. Cooper GF, Robson JG 1969b The yellow colour of the lens of man and other primates. *J. Physiol. London* **203**, 411-417.

3. Van Heyningen R 1971a Fluorescent glucoside in the human lens. *Nature* **230**, 393-394.

4. Van Heyningen R 1971b Fluorescent derivatives of 3-hydroxy-L-kynurenine in the lens of man, the baboon and the grey squirrel. *Biochem. J.* **123**, 30P-31P.

5. Van Heyningen R 1973a The glucoside of 3-hydroxykynurenine and other fluorescent compounds in the human lens. *Ciba F. Symp.* **19**, 151-171.

6. Van Heyningen R 1973b Assay of fluorescent compounds in the human lens *Exp. Eye Res.* **15**, 121-126.

7. Zigman S, Paxhia T 1988. The nature and properties of squirrel lens yellow pigment. *Exp. Eye Res.* **47,** 819-824. (doi: 10.1016/0014-4835(88)90065-6)

8. Wood AM, Truscott RJW 1993 UV filters in human lenses: tryptophan catabolism *Exp. Eye Res.* **56**, 317-325. (doi: 10.1006/exer.1993.1041)

9. Wood AM, Truscott RJW 1994 Ultraviolet filter compounds in human lenses; 3-hydroxykynurenine glucoside formation *Vision Res.* **34(11)**, 1369-1374.

(doi: 10.1016/0042-6989(94)90135-X)

10. Truscott RJW, Wood AM, Carver JA, Sheil MM, Stutchbury GM, Zhu J, Kilby GW, 1994 A new UV-filter compound in human eyes *FEBS Lett.* **348**, 173-176.

(doi: 10.1016/0014-5793(94)00601-6)

11. Snytnikova OA, Fursova, AZh, Chernyak EI, Vasiliev VG, Morozov SV, Kolosova NG, Tsentalovich YP 2008 Deaminated UV filter 3-hydroxykynureneine O-β-D-glucoside is found in cataractous human lenses *Exp. Eye Res.* **86,** 951-956.

(doi: 10.1016/j.exer.2008.03.013)

12. Hains PG, Simpanya MF, Giblin F, Truscott RJW 2006 UV filters in the lens of the thirteen lined ground squirrel (*Spermophilus tridecemlineatus*). Exp. Eye Res. **82(4)**, 730-737. (doi:10.1016/j.exer.2005.09.014).

13. Truscott RJW, Carver JA, Thorpe A, Douglas RH 1992 Identification of 3-hydroxykynurenine as the lens pigment in the gourami *Trichogaster trichopterus*. *Exp. Eye Res.* **54**, 1015-1017. (doi: 10.1016/0014-4835(92))

14. Thorpe A, Truscott RJ, Douglas RH 1992 Kynurenine identified as the short-wave absorbing lens pigment in the deep-sea fish *Stylephorus chordates*. *Exp. Eye Res.* **55**, 53-57. (doi: 10.1016/0014-4835(92)90091-6)

15. Dunlap WC, Williams DMcB, Chalker BE, Banaszak AT 1989 Biochemical photoadaptation in vision: UV-absorbing pigments in fish eye tissues. *Comp. Biochem. Physiol.* **93B**, 601-607. (doi: 10.1016/0305-0491(89)90383-0)

16. Jeon CJ, Strettoi E, Masland RH 1998 The major cell populations of the mouse retina. *J. Neurosci.*  **18(21)**, 8936-8946.

17. Mayhew TM, Astle D 1997 Photoreceptor number and outer segment disk membrane surface area in the retina of the rat: stereological data for whole organ and average photoreceptor cell. *J. Neurocytol.* **26**, 53-61.

(doi: 10.1023/A:1018563409196)

18. Glösmann M, Harlfinger PJ, Ahnelt PK (2001) S opsin-like immunoreactivity is localized to bipolar cells but not cone photoreceptors in the European hedgehog. *Invest. Ophth. Vis. Sci.* **42(4)**, S362.

19. Mowat FM, Petersen-Jones SM, Williamson H, Williams DL, Luthert PJ, Ali RR, Bainbridge JW 2008 Topographical characterisation of cone photoreceptors and the area centralis of the canine retina. *Mol. Vis.* **14**, 2518-2527.

20. Müller B, Goodman SM, Peichl L 2007. Cone photoreceptor diversity in the retinas of fruit bats (Megachiroptera). *Brain Behav. Evolut.* **70**, 90-104. (doi: 10.1159/000102971)

21. Steinberg RH, Reid M, Lacy PL 1973 The distribution of rods and cones in the retina of the cat (*Felis domesticus*). *J. Comp. Neurol.* **148**, 229-248. (doi: 10.1002/cne.901480209)

22. Jeffery G, Darling K, Whitmore A 1994 Melanin and regulation of mammalian photoreceptor topography. *Eur. J. Neurosci.* **6**, 657-667. (doi: 10.1111/j.1460-9568.1994.tb00311.x)

23. Szél Á, Röhlich P 1992 Two cone types of rat retina detected by anti-visual pigment antibodies. *Exp. Eye Res.* **55**, 47-52.

24. Chandler MJ, Smith PJ, Samuelson DA, Mackay EO 1999 Photoreceptor density of the domestic pig retina. *Vet. Ophthalmol.* **2**, 179-184.

25. Peichl L, González-Soriano J 1994 Morphological types of horizontal cell in rodent retina: A comparison of rat, mouse, gerbil and guinea pig. *Visual Neurosci.* **11**, 501-517.

26. Schiviz AN, Ruf T, Kuebber-Heiss A, Schubert C, Ahnelt KP 2008 Retinal cone topography of artiodactyl mammals: Influence of body height and habitat. *J. Comp. Neurol.* **507**, 1336-1350. (doi: 10.1002/cne.21626)

27. Krebs W, Friedrich I (1982) Quantitative morphology of bovine retina. In *The structure of the eye* (JG Hollyfield, EA Vidrio eds) Elsevier: New York pp175-182.

28. Shinozaki A, Hosaka Y, Imagawa T, Uehara M 2010 Topography of ganglion cells and photoreceptors in the sheep retina. *J. Comp. Neurol.* **518**, 2305-2315.

(doi: 10.1002/cne.22333)

29. de Farias Rocha FA, Ahnelt PK, Peichl L, Saito CA, Silveira LCL, de Lima SMA 2009 The topography of cone photoreceptors in the retina of a diurnal rodent, the agouti (*Dasyprocta aguti*). *Visual Neurosci.* **26**, 167-175.

(doi: 10.1017/S095252380808098X)

30. Young HM, Vaney DI 1991 Rod-signal interactions in the rabbit retina: Rod bipolar cells. *J. Comp. Neurol.* **310**, 139-153. (doi: 10.1002/cne.903100202)

31. Francois J, Wouters L, Victoriatroncoso V, Derouck A, Vangerven A 1980 Morphometric and electrophysiological study of the photoreceptors in the horse. *Ophthalmologica* **181(6)**, 340-349.

32. Sandmann D, Boycott BB, Peichl L 1996 Blue-cone horizontal cells in the retinae of horses and other *Equidae*.  *J. Neurosci.* **16(10)**, 3381-3396.

33. Solovei I, Kreysing M, Lanctot C, Kosem S, Peichl L, Cremer T, Guck J, Joffe B 2009 Nuclear architecture of rod photoreceptor cells adapts to vision in mammalian evolution. *Cell* **137(2)**, 356-368. (doi: 10.1016/j.cell.2009.01.052)

34. Finlay BL, Franco ECS, Yamada ES, Crowley JC, Parsons M, Augusto J, Muniz PC, Silveira LCL 2008 Number and topography of cones, rods and optic nerve axons in New and Old World primates. *Visual Neurosci.* **25**, 289-299.

(doi: 10.1017/S0952523808080371)

35. Kryger Z, Galli-Resta l, Jacobs GH, Reese BE 1998 The topography of rod and cone photoreceptors in the retina of the ground squirrel. *Visual Neurosci.* **15**, 685-691

36. Clark D, Hendrickson A, Curcio C 1988 Photoreceptor topography in lid-sutured *Macaca fasicularis*. *Invest. Ophth. Vis. Sci.* **29**, 33.

37. Wikler KC, Williams RW, Rakic P (1990) Photoreceptor mosaic: Number and distribution of rods and cones in the Rhesus monkey retina. *J. Comp. Neurol.* **297**, 499-508. (doi: 10.1002/cne.902970404)

38. Müller B, Peichl L 1989 Topography of cones and rods in the tree shrew retina. *J. Comp. Neurol.* **282**, 581-594. (doi: 10.1002/cne.902820409)

39. West RW, Dowling JE 1975 Anatomical evidence for cone and rod-like receptors in the gray squirrel, ground squirrel, and prairie dog retinas. *J. Comp. Neurol.* **159**, 439-460. (doi: 10.1002/cne.901590402)
